# The role of N(6)-methyladenosine (m6a) modification in cancer: recent advances and future directions

**DOI:** 10.17179/excli2024-7935

**Published:** 2025-01-15

**Authors:** Xiaozhou Xie, Zhen Fang, Haoyu Zhang, Zheng Wang, Jie Li, Yuchen Jia, Liang Shang, Feng Cao, Fei Li

**Affiliations:** 1 Department of General Surgery, Xuanwu Hospital, Capital Medical University, Beijing, China; 2 Department of Gastrointestinal Surgery, Shandong Provincial Hospital Affiliated to Shandong First Medical University, Jinan, Shandong, China

**Keywords:** N(6)-methyladenosine (m6A), writers, erasers, readers, cancer

## Abstract

N(6)-methyladenosine (m6A) modification is the most abundant and prevalent internal modification in eukaryotic mRNAs. The role of m6A modification in cancer has become a hot research topic in recent years and has been widely explored. m6A modifications have been shown to regulate cancer occurrence and progression by modulating different target molecules. This paper reviews the recent research progress of m6A modifications in cancer and provides an outlook on future research directions, especially the development of molecularly targeted drugs.

See also the graphical abstract(Fig. 1).

## Introduction

Post-transcriptional modification of RNA is a key component of epigenetics, and to date, more than 170 identified RNA modifications, including RNA methylation, have been identified (Roundtree et al., 2017[203]). In the 1970s, adenosine was shown to be methylated at the nitrogen atom of N(6), forming N(6)-methyladenosine (m6A) (Desrosiers et al., 1974[46]). The m6A modification mainly occurs on the adenine in the RRACH sequence, with significant enrichment at the 3'UTR and near stop codons, and its modification is dynamically reversible (Meyer et al., 2012[172]). Currently, m6A modifications have been identified as the most abundant and prevalent internal modifications in eukaryotic mRNAs (Jiang et al., 2021[109]). With the development of Next-generation sequencing, breakthroughs have been made in the role of m6A modifications in eukaryotes (Tavakoli et al., 2023[236]; Chen et al., 2015[21]). m6A modifications are closely associated with almost all aspects of RNA-related biological processes, including transcription, precursor mRNA splicing and processing, nuclear export, translation, RNA stability and decay (Lesbirel et al., 2018[120]; Wang et al., 2014[274], 2022[266]). In addition to this, m6A modifications are also involved in other biological processes, such as transcriptional regulation and signal transduction (Zhang et al., 2024[337]; Patil et al., 2016[188]; Lee et al., 2021[118], 2021[119]). m6A dysregulation contributes to the development of a wide range of human diseases. Notably, m6A modifications play an important regulatory role in the occurrence and development of human cancer. It has been found that m6A regulates cancer progression through its involvement in the regulation of autophagy (Yu et al., 2024[324]), cell cycle (Xu et al., 2024[304]; Xia et al., 2024[299]), DNA damage (Cesaro et al., 2024[12]), ferroptosis (Wu et al., 2023[296]), chemotherapeutic resistance (Zhou et al., 2024[364]), and oncogenes/anti-oncogenes expression (Wang et al., 2024[279], 2023[273]). This review summarizes the research progress of m6A modification in cancer and looks into the future trends and possible research directions of m6A modification.

## The Writers, Erasers, and Readers of m6A

Enzymes are involved in the exercise of m6A function, including methylation transferase (writer), demethylation enzyme (eraser) and methylation recognition protein (reader) (Yang et al., 2018[318]). Writers include METTL3/4/5/14/16, WTAP, VIRMA, RBM15, RBM15B, ZC3H13 and ZCCHC4. Erasers include FTO, ALKBH5. Readers include IGF2BP1/2/3, YTHDF1/2/3, YTHDC1/2, HNRNPC, HNRNPG, HNRNPA2B1, FMRP, and PRRC2A. These m6A regulators play different roles (Table 1(Tab. 1); References in Table 1: Alarcón et al., 2015[1]; Bokar et al., 1997[8]; Chang et al., 2020[13]; Chen et al., 2020[17], 2022[28]; Goh et al., 2020[62]; Hsu et al., 2017[81], 2019[80]; Huang et al., 2018[91], 2021[94]; Liu et al., 2014[151], 2015[154]; Patil et al., 2016[188]; Pendleton et al., 2017[189]; Ping et al., 2014[191]; Ren et al., 2019[199], 2023[200]; Roundtree et al., 2017[204]; Sepich-Poore et al., 2022[205]; Shi et al., 2017[211]; Wang et al., 2014[274], 2015[276], 2016[259]; Warda et al., 2017[280]; Wei et al., 2018[282]; Wen et al., 2018[286]; Wu et al., 2019[292], 2024[294]; Xiao et al., 2016[300]; Yue et al., 2018[329]; Zhang et al., 2018[339]; Zhao et al., 2020[357]; Zheng et al., 2013[358]; Zhou et al., 2019[362]), and their dysregulation or aberrant expression affects cancer progression and thus the clinical prognosis of cancer patients.

## Writers

METTL3, a core component of the m6A methyltransferase complex (MTC), was first characterized in 1997 (Bokar et al., 1997[8]). METTL3 and METTL14 form a heterodimeric complex that co-catalyzes m6A modification, with METTL3 being the catalytic subunit that binds S-adenosylmethionine (SAM), and METTL14 playing a crucial structural role in substrate recognition (Wang et al., 2016[259]; Liu et al., 2014[151]). METTL3 plays a pro-carcinogenic role in most malignant tumors (Cheng et al., 2024[33]; Bhattarai et al., 2024[6]; Vaid et al., 2024[239]), but also acts as a cancer suppressor in certain tumors (Chen et al., 2024[26]; Zhang et al., 2024[335]). METTL14 also exerts oncogenic and anticancer effects in different tumors. METTL14 promotes cell proliferation of myeloid proliferative neoplasms by regulating SETBP1 (Jiang et al., 2024[106]). METTL14 downregulates CircUGGT2 to inhibit the progression of gastric cancer (Chen et al., 2024[29]).

WTAP does not have methylation activity, but its interaction with the METTL3-METTL14 complex is required for their localization to nuclear speckles enriched in pre-mRNA processing factors (Liu et al., 2014[151]; Ping et al., 2014[191]). WTAP affects tumor progression by regulating cell cycle (Jin et al., 2024[114]), mitophagy (Wang et al., 2024[254]), production of reactive oxygen species (Ji et al., 2024[99]), oxidative phosphorylation (Jia et al., 2023[104]), ferroptosis (Tan et al., 2024[231]; Wang et al., 2023[256]), and chemotherapy drug resistance (Wei et al., 2021[283]).

METTL4 is a methyltransferase of U2 snRNA that regulates RNA splicing (Chen et al., 2020[17]; Goh et al., 2020[62]). Currently, it has been found that METTL4 affects tumor development by regulating ferroptosis (Liu et al., 2023[158]; Shen et al., 2021[209]). 

METTL5 catalyzes the m6A modification of nucleotide A-1832 in human 18S rRNA (van Tran et al., 2019[240]). It contains one active site, one substrate binding site, and one catalytic site (van Tran et al., 2019[240]; Turkalj and Vissers, 2022[238]). The research on METTL5 in cancer has emerged in recent years, and current research is mostly focused on the field of hepatocellular carcinoma (Xia et al., 2023[298]; Wang and Peng, 2023[257]; Luo et al., 2024[164]; Wang et al., 2024[262]; Xu et al., 2023[306]). 

The methylation substrates of METTL16 must meet specific sequential and structural requirements (Doxtader et al., 2018[51]; Mendel et al., 2018[171]), so the abundance of substrates for METTL16 is very low, and so far only two substrates, the MAT2A transcript encoding SAM synthase and the U6 snRNA, have been confirmed (Pendleton et al., 2017[189]; Warda et al., 2017[280]).

VIRMA (KIAA1429) is located in the nuclear speckles (Zhu et al., 2021[375]), and as the largest known component of the MTC (202 kDa), it participates in the formation of the MTC and acts as a scaffold, and recruits the m6A complex to specific RNA sites (Yue et al., 2018[329]). VIRMA was found to promote hepatocellular carcinoma progression by regulating the m6A modification of GATA3 (Lan et al., 2019[116]) and lung adenocarcinoma by regulating BTG2 (Zhang et al., 2022[338]) and JNK/MAPK pathways (Lin et al., 2023[146]).

RBM15 collaborates with its analog RBM15B to recruit MTC to specific sites of long noncoding RNA X inactivation-specific transcript (XIST) and promote XIST-mediated gene silencing (Patil et al., 2016[188]). In recent years, the research results of RBM15 in cancer have been remarkable, and it has been found that RBM15 acts as an oncogene in breast cancer (Park et al., 2024[187]), laryngeal cancer (Wang et al., 2021[275]), pancreatic cancer (Dong et al., 2023[49]), bladder cancer (Huang et al., 2024[95]), esophageal squamous carcinoma (Wang, 2024[245]), renal clear cell carcinoma (Zeng et al., 2022[330]), cervical cancer (Song and Wu, 2023[216]), and ovarian cancer (Yuan et al., 2023[326]).

ZC3H13 regulates m6A methylation by inducing nuclear localization of ZC3H13-WTAP-Vrilizer-Hakai complex (Zhao et al., 2020[357]; Wen et al., 2018[286]). ZC3H13 plays dual roles in different tumors. ZC3H13 enhances cervical cancer stemness and chemotherapy resistance by promoting m6A modification of CENPK (Lin et al., 2022[145]). ZC3H13 inhibits the progression of colorectal cancer by suppressing the Ras-ERK pathway (Zhu et al., 2019[371]). ZC3H13 mediates m6A modification of PHF10 to induce a DNA damage response to promote pancreatic cancer that can be inhibited by fisetin (Huang et al., 2022[87]).

ZCCHC4 is a 28S rRNA specific m6A methyltransferase (Ren et al., 2019[199]), but there are few reports on its role in tumors. ZCCHC4 promotes chemotherapy resistance in hepatocellular carcinoma by disrupting DNA damage-induced apoptosis (Zhu et al., 2022[372]). Additionally, it facilitates the development of colorectal cancer via the ZCCHC4-LncRNA GHRLOS-KDM5D axis (Ma et al., 2019[169]).

## Erasers

FTO was identified as the first m6A demethylase in 2011 (Jia et al., 2011[102]). FTO can bind to various RNAs, including mRNA, snRNA, and tRNA, and can demethylate the internal m6A and cap m6Am in mRNA (Wei et al., 2018[282]). In the past 5-10 years, research on the impact of FTO on cancer progression has begun to emerge. FTO promotes the tumorigenesis of hepatocellular carcinoma and suppresses tumor immunity (Chen et al., 2024[15]). FTO fosters the tumorigenesis of colorectal cancer by triggering the expression of SLC7A11/GPX4 (Qiao et al., 2024[195]). FTO promotes tumor progression in gastric cancer (Zeng et al., 2024[331]; Wu et al., 2024[295]), bladder cancer (Wu et al., 2024[290]), colorectal cancer (Qiao et al., 2024[195]), hepatocellular carcinoma (Chen et al., 2024[15]; Jiang et al., 2024[105]), lung cancer (Gao et al., 2023[58]), cervical cancer (Wang et al., 2023[242]), and pancreatic cancer (Tan et al., 2022[233]; Wang et al., 2023[269]). FTO exerted a tumor-suppressing effect in thyroid cancer (Huang et al., 2022[92]; Ji et al., 2022[98]) and cholangiocarcinoma (Gao et al., 2021[59]; Rong et al., 2019[202]). However, in prostatic cancer (Zhao et al., 2024[355]; Hu et al., 2024[82]), clear cell renal cell carcinoma (Xu et al., 2022[310]; Shen et al., 2022[208]; Strick et al., 2020[218]; Zhuang et al., 2019[378]), and breast cancer (Xu et al., 2020[309]; Ni et al., 2024[177]; Ou et al., 2022[182]; Yan et al., 2024[312]), FTO has a dual pro-cancer and anti-carcinogenic role, or the current role is controversial.

ALKBH5 is another m6A demethylase. The demethylation activity of ALKBH5 affects mRNA output and RNA metabolism, as well as the assembly of mRNA processing factors in nuclear spots (Zheng et al., 2013[358]). The role of ALKBH5 in cancer has been widely demonstrated, and it affects cancer progression by regulating various biological processes such as proliferation, migration, invasion, and metastasis (Wang et al., 2020[251]). Recent studies have found that ALKBH5 positively correlates with PD-L1 expression and macrophage infiltration and promotes non-small cell lung cancer progression by regulating tumor immunity through JAK2/p-STAT3 (Hua et al., 2024[85]). ALKBH5 reduces CD58 in gastric cancer cells through m6A methylation, activates the PD-1/PD-L1 axis, and ultimately induces immune escape from gastric cancer cells (Suo et al., 2024[228]). ALKBH5 drives immunosuppression by targeting AXIN2 to promote colorectal cancer (Zhai et al., 2023[333]). Furthermore, ALKBH5 promotes the progression of ovarian cancer (An and Duan, 2024[2]), colorectal cancer (Sun et al., 2024[221]), and lung adenocarcinoma (Tan et al., 2024[230]) by regulating macrophage polarization. The above suggests that ALKBH5 plays an important role in mediating tumor immunity and regulating the tumor microenvironment, and is an important potential target for immunotherapy of malignant tumors.

## Readers

IGF2BP1/2/3 are members of the Insulin-like growth factor-2 mRNA-binding proteins (IGF2BPs) family, and IGF2BPs are a highly conserved family of RNA-binding proteins (Nielsen et al., 1999[179]; Zhu et al., 2023[374]). In 2018, Huang et al. demonstrated that IGF2BP1/2/3 act as new m6A reader family members, and that IGF2BPs contribute to the stability and translation of thousands of potential mRNA targets in an m6A-dependent manner, thereby affecting gene expression (Huang et al., 2018[91]). IGF2BPs are overexpressed in various cancers, and recent studies have found that IGF2BP1 interacts with RPS15 and promotes the development of esophageal squamous cell carcinoma by recognizing m6A modifications (Zhao et al., 2023[356]). IGF2BP2 promotes cell cycle progression in triple-negative breast cancer through recruitment of EIF4A1 (Xia et al., 2024[299]). IGF2BP3 binds the SENP1 3-UTR in an m6A manner and enhances SENP1 expression, which in turn exacerbates acute myeloid leukemia (Wen et al., 2024[285]).

YTHDF1/2/3 and YTHDC1/2 all contain YT521-B homology (YTH) structural domains (Nayler et al., 2000[176]; Hartmann et al., 1999[74]). The YTH structural domain is an RNA-binding structural domain specialized for m6A recognition (Zhang et al., 2010[353]; Zou and He, 2024[379]). YTHDF1 is the most abundant m6A reader, which promotes protein translation (Ren et al., 2023[200]). YTHDF1 exhibited carcinogenic effects in colorectal cancer (Chen et al., 2024[16]), esophageal cancer (Zhang et al., 2024[342]), breast cancer (Wang et al., 2024[267]), gastric cancer (Song et al., 2024[215]), gallbladder cancer (Chen et al., 2024[18]), non-small cell lung cancer (Sun et al., 2024[225]), hepatocellular carcinoma (Zhang et al., 2024[349]), and bladder cancer (Zhu et al., 2023[373]). YTHDF2 regulates RNA degradation (Chen et al., 2022[28]; Hsu et al., 2017[81]), and YTHDF2 is a oncogenic gene in most cancer types (Bai et al., 2023[3]; Jin et al., 2024[111]; Jiang et al., 2024[107]; Li et al., 2020[128]; Zhang et al., 2023[343]), however, it exerted both carcinogenic and anticarcinogenic effects in gastric cancer (Fang et al., 2023[55]; Ren et al., 2024[198]; Zhou et al., 2023[366]), hepatocellular carcinoma (Yang et al., 2023[319]; Wen et al., 2024[287]; Hou et al., 2019[78]; Zhong et al., 2019[360]), and pancreatic cancers (Tan et al., 2022[233]; Guo et al., 2020[66]). YTHDF3 enhances mRNA translation assisted by YTHDF1 (Ren et al., 2023[200]; Chang et al., 2020[13]; Shi et al., 2017[211]), which is currently less studied in the field of oncology, and acts as an oncogene similar to YTHDF1 (Chang et al., 2020[13]; Zhang et al., 2024[340]; Duan et al., 2024[53]). YTHDC1 regulates mRNA splicing by recruiting and modulating pre mRNA splicing factors, enabling it to enter the binding region of targeted mRNA, and mediate the nuclear export of methylated mRNA (Xiao et al., 2016[300]; Roundtree et al., 2017[204]). YTHDC2 can improve the translation efficiency of target mRNA and also reduce mRNA abundance (Hsu et al., 2017[81]; Wu et al., 2024[294]). YTHDC1/2 have carcinogenic and anticarcinogenic effects in different cancers (Yuan et al., 2022[328], 2023[327]; Tan et al., 2022[229]; Yan et al., 2023[311]; Hou et al., 2019[79]; Zhou and Wang, 2024[361]; Wang et al., 2021[249], 2022[250]). 

HNRNPC, HNRNPG, and HNRNPA2B1 are members of the HNRNP proteins. HNRNP (heterogeneous nuclear ribonucleo protein) can participate in multiple RNA metabolic processes, and its most widely studied function is to participate in RNA splicing processes (Zhang et al., 2021[346]). HNRNPC mediates mRNA splicing, 3'-terminal processing, and translation (Huang et al., 2021[94]; Liu et al., 2015[154]). Recent studies have found that circPPAP2B promotes proliferation and metastasis of renal clear cell carcinoma through HNRNPC-dependent alternative splicing (Zheng et al., 2024[359]). HNRNPC also functions as an oncogene in other cancers (Huang et al., 2024[93]; Chen et al., 2024[20]; Lian et al., 2023[140]). HNRNPG uses the Arg-Gly-Gly (RGG) motif to selectively bind m6A-modified RNA and regulate selective splicing (Zhou et al., 2019[362]). Current studies have only found potential effects on endometrial cancer (Hirschfeld et al., 2015[77]). HNRNPA2B1 is a nuclear reader of m6A and mediates effects on primary microRNA processing and selective splicing (Alarcón et al., 2015[1]). HNRNPA2B1 is also an m6A reader that drives cancer progression (Wang et al., 2024[253]; Yu et al., 2024[325]; Jin et al., 2024[113]; Liu et al., 2022[148]).

FMRP is one of the readers of m6A, which may affect translation directly or through interaction with YTHDF proteins (Wang et al., 2014[274], 2015[276]; Zhang et al., 2018[339]; Hsu et al., 2019[80]). And FMRP can affect nuclear mRNA output by recognizing m6A-modified mRNAs (Hsu et al., 2019[80]). Research has found that METTL3 mediated m6A modified FMRP drives the progression of hepatocellular carcinoma (Fu et al., 2024[56]). PRRC2A is a newly discovered m6A reader in 2019 that regulates the stability of its target Olig2 mRNA by specifically binding methylated RNA through the GRE structural domain (Wu et al., 2019[292]). PRRC2A has not yet been reported to function as an m6A reader in cancer.

## m6A Modification in Cancer

**Figure 2:**
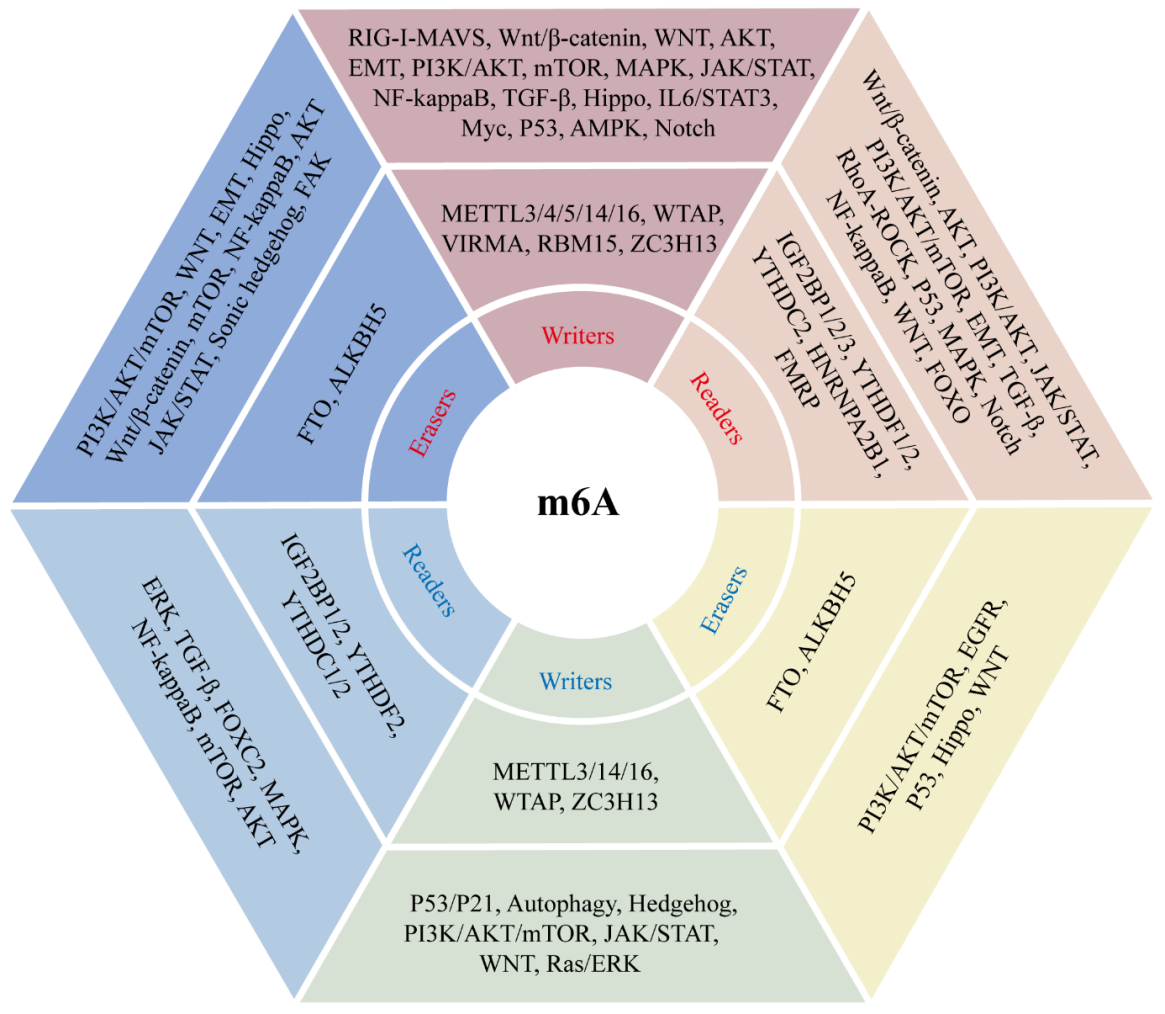
Signaling pathways regulated by m6A modification regulators. Red color indicates m6A regulators with oncogenic role, blue color indicates m6A regulators with anti-oncogenic role.

**Figure 3:**
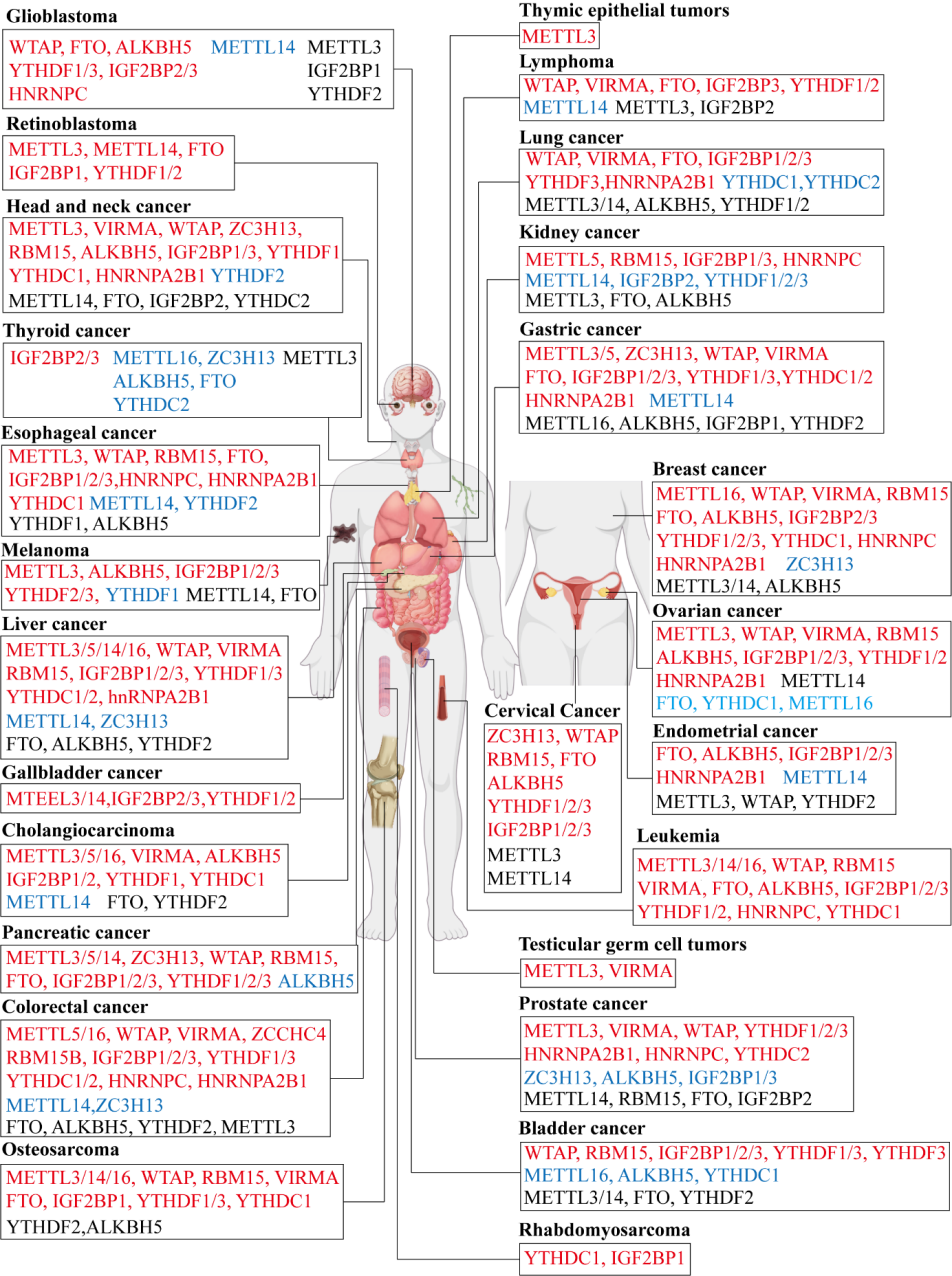
The role of m6A modification regulators in cancer. Red color indicates oncogenic regulators, blue color indicates anti-oncogenic regulators, and black color indicates regulators that have both oncogenic and anti-oncogenic effects.

Studies in recent years have illustrated that m6A modifications are strongly associated with cancer progression. And m6A modification regulators affect cancer progression by regulating different signaling pathways (Figure 2(Fig. 2)). We analyzed the effects of m6A modifications on the occurrence and progression of different cancers, starting from different cancer types (Figure 3(Fig. 3)).

## Breast Cancer

Breast cancer has been reported to have surpassed lung cancer as the most common cancer among women and is the leading cause of cancer-related deaths among women (Bray et al., 2024[9]). Majority of m6A modifications promote breast cancer occurrence and progression. METTL16 regulates the mRNA stability of FBXO5 via m6A modification, thereby promoting the malignant behavior of breast cancer (Wang et al., 2024[263]). YTHDF1 promotes osteolytic bone metastasis in breast cancer by inducing translation (Wang et al., 2024[267]). HNRNPA2B1 promotes breast cancer progression by regulating mRNA selective export (Jin et al., 2024[113]). In contrast, ZC3H13 was found to be a tumor suppressor gene in breast cancer (Gong et al., 2020[63]). In addition, METTL3, METTL14, and ALKBH5 have been reported in studies of promoting and inhibiting breast cancer, suggesting that they may play both oncogenic and anti-oncogenic roles in breast cancer (Gong et al., 2020[63]; Li et al., 2024[138]; Xu et al., 2023[308]; Wang et al., 2024[247]; Sun et al., 2020[224]; Woodcock et al., 2024[288]; Liu et al., 2022[149]).

## Lung Cancer

Lung cancer remains the most commonly diagnosed cancer in the entire population (12.4 % of all cancers globally) and is the leading cause of cancer deaths (18.7 % of all cancers) (Bray et al., 2024[9]). m6A modifications are strongly associated with lung cancer progression and chemotherapy resistance. In lung cancer, the m6A writer METTL3 has been most widely and intensively studied. METTL3 can promote chemoresistance in small cell lung cancer by inducing mitochondrial autophagy (Sun et al., 2023[226]). HIF-1α drives smoking-induced non-small cell lung cancer progression by promoting cell proliferation through METTL3-regulated m6A modification (Yang et al., 2023[317]). However, it has been shown that METTL3 is downregulated in lung cancer tissues and inhibits the migration and invasive ability of lung cancer cells in a YTHDF1-dependent manner (Zhang et al., 2024[342]). Although METTL3 has been extensively studied in lung cancer, its expression and role in lung cancer are still controversial and further studies are needed in the future. In addition to METTL3, METTL14, ALKBH5, and YTHDF1/2 also exhibited both oncogenic and anti-oncogenic effects in lung cancer (Gao et al., 2023[58]; Hua et al., 2024[85]; Ji et al., 2024[101]; Li et al., 2022[125]; Sun et al., 2022[227]; Tsuchiya et al., 2021[237]; Dou et al., 2022[50]). Furthermore, we found that among the m6A readers, the IGF2BPs family was closely associated with pro-tumor progression in lung cancer (Sun et al., 2023[220]; Zhou et al., 2024[370]; Lin et al., 2023[147]), while YTHDC1/2 was a suppressor gene of lung cancer progression (Yuan et al., 2023[327]; Sun et al., 2020[223]), and may become therapeutic targets for lung cancer.

## Thyroid Cancer

Thyroid cancer is the seventh most common cancer and the fifth most common in women, with three times the incidence in women than in men, but with a lower mortality rate (Bray et al., 2024[9]). Existing literature indicates that m6A modification regulators mainly play an anti-cancer role in thyroid cancer. ZC3H13 increased the m6A modification of hsa_circ_0101050 and inhibited its expression, which in turn inhibited thyroid cancer. ZC3H13 increases the m6A modification of hsa_circ_0101050 and inhibits its expression, thereby suppressing thyroid cancer (Lv et al., 2024[167]). Demethylase ALKBH5 reduces m6A modification of circNRIP1 and down-regulates its expression to inhibit glycolysis in thyroid cancer cells (Ji et al., 2023[100]). YTHDC2 inhibits proliferation and induces apoptosis in thyroid cancer cells by regulating CYLD-mediated inactivation of the Akt pathway (Zhou and Wang, 2024[361]). FTO inhibits glycolysis and growth of thyroid cancer cells by destabilizing APOE mRNA with m6A modification (Huang et al., 2022[92]). METTL16 attenuates lipid metabolism via m6A-mediated stabilization of SCD1 mRNA and thus inhibits thyroid cancer (Li et al., 2024[134]). In contrast, IGF2BP2/3 exerts oncogenic effect in thyroid cancer (Wang et al., 2024[268]; Panebianco et al., 2017[186]). However, METTL3 has oncogenic and anti-oncogenic roles in thyroid cancer (Ning et al., 2023[180]; Lin et al., 2022[144]). 

## Gastric Cancer

Data from 2022 show that there are more than 968,000 new cases of gastric cancer globally, the fifth highest incidence and mortality rate among all cancers (Bray et al., 2024[9]). It was found that m6A plays an important role in the progression of gastric cancer. m6A modification promotes the proliferation (Wu et al., 2024[295]; Li et al., 2024[137]; Xu et al., 2022[305]) and metastasis (Liu et al., 2022[159]; Wang et al., 2024[255]) of gastric cancer, inhibits cell ferroptosis (Niu et al., 2024[181]; Yang et al., 2022[314]) and apoptosis (Ci et al., 2024[35]), and promotes chemotherapy resistance (Wang et al., 2024[253]; Zhu et al., 2022[376]) and immune escape (Suo et al., 2024[228]; Tang et al., 2024[234]) in gastric cancer. m6A modification usually promotes gastric cancer progression. IGF2BP1 recognizes METTL3-mediated m6A modification of APAF1-binding lncRNA (ABL), which enhances ABL stability and thus promotes gastric cancer proliferation and chemoresistance (Wang et al., 2022[260]). METTL5 promotes NRF2 mRNA stability, which in turn inhibits ferroptosis and promotes immune escape in gastric cancer (Li et al., 2024[136]). Acetylated SRSF2 binds YTHDF1 pre-mRNA, leading to enhanced YTHDF1 exon 4 skipping, which stimulates GC cell proliferation and migration (Liu et al., 2024[156]). Among the available studies, only METTL14 among the m6A modification regulators showed complete tumor suppression in gastric cancer. METTL14 mediates m6A modification of circORC5 to inhibit gastric cancer progression by regulating miR-30c-2-3p/AKT1S1 axis (Fan et al., 2022[54]). METTL14 also mediates m6A modification of circUGGT2 to inhibit gastric cancer progression and chemoresistance by regulating the miR-186-3p/MAP3K9 axis (Chen et al., 2024[29]). Unlike most m6A modification regulators, ALKBH5 (Suo et al., 2024[228]; Hu et al., 2022[83]; Wang et al., 2024[261]), IGF2BP1 (Xu et al., 2022[305]; Ding et al., 2024[47]), and YTHDF2 (Ren et al., 2024[198]; Yang et al., 2022[314]; Shen et al., 2020[210]) exhibit both oncogenic and anti-oncogenic effects in gastric cancer. 

## Colorectal Cancer

According to 2022 data, there were more than 1.9 million new cases of colorectal cancer and 904,000 deaths, and that colorectal cancer had the third highest incidence but the second highest mortality rate (Bray et al., 2024[9]). Similar to other tumors, the regulation of m6A modifications in colorectal cancer is equally diverse and complex, and almost all m6A modifiers promote colorectal carcinogenesis. Similar to other tumors, the regulation of m6A modifications in colorectal cancer is equally diverse and complex, and almost all m6A modifiers except METTL14, ZC3H13, METTL3, FTO, ALKBH5, and YTHDF2 promote colorectal carcinogenesis. METTL14 and ZC3H13 play important regulatory roles in inhibiting colorectal cancer proliferation and metastasis (Zhu et al., 2019[371]; Chen et al., 2020[27]; Yang et al., 2020[316]). METTL3 (Ouyang et al., 2024[184]; Jiang et al., 2024[108]; Deng et al., 2019[45]), FTO (Qiao et al., 2024[195]; Ye et al., 2023[322]), ALKBH5 (Zhai et al., 2023[333]; Ye et al., 2023[322]), and YTHDF2 (Qiao et al., 2024[195]; Chen et al., 2020[27]; Shen et al., 2023[207]) may have different mechanisms to exhibit oncogenic and anti-oncogenic roles in colorectal cancer. 

## Liver Cancer

Primary liver cancer consists mainly of hepatocellular carcinoma (75 %-85 %) and intrahepatic cholangiocarcinoma (10 %-15 %) (Bray et al., 2024[9]; de Martel et al., 2020[43]). The incidence of liver cancer has been steadily decreasing as the number of HBV and HCV positive people has declined and aflatoxin exposure has decreased, but liver cancer remains the third leading cause of cancer death after lung cancer and colorectal cancer (Bray et al., 2024[9]). Considerable research has been published on m6A modification in hepatocellular carcinoma, and m6A modifiers mainly act as oncogenic factors. In addition to ZC3H13 and METTL14, m6A writers have been shown to be oncogenic factors in liver cancer. METTL3-mediated m6A modification leads to the upregulation of TUG1, which interacts with YBX1 to promote the upregulation of PD-L1 and CD47 transcripts, ultimately regulating tumor immune escape (Xi et al., 2024[297]). METTL16 regulates SENP3 mRNA stability in an m6A-dependent manner, confers ferroptosis resistance and promotes tumor progression in hepatocellular carcinoma (Wang et al., 2024[252]). In contrast, m6A writers METTL14 and ZC3H13 inhibit liver cancer progression. USP48 is regulated by METTL14-induced m6A modification and stabilizes SIRT6 to attenuate hepatocellular carcinoma glycolysis and inhibit progression (Du et al., 2021[52]). ZC3H13 is lowly expressed in hepatocellular carcinoma and may be involved in transcriptional dysregulation or the JAK-STAT pathway to inhibit tumor migration and invasion (Wu et al., 2022[293]). m6A readers also act as oncogenic factors in liver cancer in addition to YTHDF2. YTHDF1 promotes stemness and treatment resistance in hepatocellular carcinoma by enhancing NOTCH1 expression (Zhang et al., 2024[349]). Positive functional loops of YTHDF3 and PFKL in glucose metabolism in hepatocellular carcinoma promote tumor proliferation and metastasis (Zhou et al., 2022[363]). Whereas the role of YTHDF2 and m6A erasers FTO and ALKBH5 in hepatocellular carcinoma remains unclear or different mechanisms exist to promote and suppress tumors (Chen et al., 2022[30], 2024[15]; Zhong et al., 2019[360]; Liao et al., 2023[142]; Liu et al., 2022[152]; Wang et al., 2023[271]). 

## Cholangiocarcinoma

According to different locations, cholangiocarcinoma is classified into intrahepatic, perihilar and distal cholangiocarcinoma, and has a lower incidence compared to hepatocellular carcinoma (Brindley et al., 2021[10]). The incidence of cholangiocarcinoma is increasing every year, but its global epidemiology varies widely (Montal et al., 2020[175]). m6A modification in cholangiocarcinoma remains understudied at present. The current study suggests that METTL proteins are closely related to the pro-carcinogenic effects of cholangiocarcinoma. METTL3 mediates m6A modification of circSLCO1B3 and promotes intrahepatic cholangiocarcinoma proliferation and metastasis via miR-502-5p/HOXC8/ SMAD3 axis (Li et al., 2024[129]). METTL5-mediated m6A modification of 18S rRNA promotes growth and metastasis of intrahepatic cholangiocarcinoma cells (Dai et al., 2023[41]). METTL16 regulates FGFR4 expression in cholangiocarcinoma cells through PRDM15 signaling and promotes tumor proliferation and progression (Liu et al., 2023[155]). In contrast METTL14-mediated m6A modification inhibits the MACF1/β-catenin pathway in cholangiocarcinoma, which in turn exerts tumor suppressor effects (Zhang et al., 2022[352]). 

## Prostate Cancer

Prostate cancer is the second most common cancer in the world, the most commonly diagnosed cancer in nearly two-thirds of men worldwide, and the fifth leading cause of cancer deaths in men (Bray et al., 2024[9]). In prostate cancer, METTL3 remains the most studied m6A modification regulators, and METTL3 mediates m6A modification of USP4 mRNA at A2696 to promote prostate cancer invasion and metastasis (Chen et al., 2021[31]). m6A reader also plays an important regulatory role in prostate cancer. YTHDF1/2/3 promote prostate cancer proliferation, invasion and metastasis and suppress anti-tumor immunity by different mechanisms (Li et al., 2020[128], 2022[132]; Duan et al., 2024[53]; Wang et al., 2024[277]). HNRNP proteins are also cancer promoters in prostate cancer. HNRNPA2B1 induces maturation of miR-25-3p/miR-93-5p to regulate TGF-β and FOXO pathways leading to prostate cancer progression (Qi et al., 2023[194]). HNRNPC suppresses tumor immunity by increasing Treg cell activation and suppressing CD8 T cells (Cheng et al., 2023[34]). In contrast to HNRNP proteins, IGF2BPs cause elevated overall R-loop levels, cell migration and growth inhibition in prostate cancers by preventing DNMT1 binding to the SEMA3F promoter (Ying et al., 2024[323]). However, it has also been shown that IGF2BP2 is recruited by circABCC4, enhances CCAR1 mRNA stability and activates the Wnt/β-catenin pathway to promote prostate cancer stemness and metastasis (Huang et al., 2023[86]). 

## Bladder Cancer

Bladder cancer is the ninth most commonly diagnosed cancer in the world and is far more common in men than in women, but even among women it is the sixth most common cancer and the ninth leading cause of cancer deaths (Bray et al., 2024[9]). Recent studies have shown that m6A writer WTAP and circ0008399 interactions promote MTC assembly and activity and cisplatin resistance in bladder cancer (Wei et al., 2021[283]). For METTL3, studies have shown that it and RBM15 synergistically mediate m6A modification of lncRNAs to promote malignant progression of bladder cancer (Huang et al., 2024[95]). Moreover, METTL3 promotes tumor proliferation in bladder cancer by accelerating the maturation of pri-miR221/222 in an m6A-dependent manner (Han et al., 2019[70]). However, other studies have shown that METTL3 overexpression enhances m6A modification of LINC01106 in bladder cancer cells and inhibits tumor progression (Liu et al., 2024[150]). m6A reader similarly regulates bladder cancer progression. IGF2BPs are pro-cancer factors in bladder cancer and promote tumor proliferation and metastasis (Xie et al., 2021[301]; Tan et al., 2024[232]; Lv et al., 2024[168]). Similar to the IGF2BPs family, YTHDF1/2/3 promote bladder cancer progression and suppress tumor immunity (Zhang et al., 2023[343]; Jin et al., 2019[112]; Qiu et al., 2024[196]). However, the study by Zeng et al. also found that YTHDF2 degrades DHCR7 mRNA and inhibits cholesterol synthesis and cAMP signaling, which in turn inhibits bladder cancer metastasis (Zeng et al., 2024[332]). 

## Esophageal Cancer

Esophageal cancer is the 11th most commonly diagnosed cancer and the 7th leading cause of cancer deaths worldwide (Bray et al., 2024[9]). METTL3 promotes the proliferation, invasion and metastasis of esophageal cancer by regulating the methionine cycle (Jin et al., 2024[115]), Wnt/β-catenin (Zhang et al., 2024[348]), EMT (Wu et al., 2024[289]), PI3K/AKT (Jia and Yu, 2024[103]), Notch (Han et al., 2021[69]) signaling pathways and glycolysis (Gao et al., 2023[60]). m6A reader IGF2BPs family members similarly contribute to the malignant progression of esophageal cancer. IGF2BP1 promotes translation of p38 MAPK pathway proteins by recognizing and directly binding to the mRNAs of MKK6 and MAPK14 (Zhao et al., 2023[356]). IGF2BP2 induces circRUNX1 with m6A modification and promotes esophageal cancer proliferation and metastasis via miR-449b-5p/FOXP3 axis (Wang et al., 2022[244]). And linc01305 was found to promote esophageal cancer progression by interacting with IGF2BP2 and IGF2BP3 (Huang et al., 2021[88]). HNRNP proteins are also cancer-promoting factors in esophageal cancer (Li et al., 2021[130]; Zhou et al., 2023[368]). Differently, YTHDF2 and METTL14 exhibit anticancer effects in esophageal cancer (Cui et al., 2021[38]; Liu et al., 2021[162]). Furthermore, YTHDF1 and ALKBH5 have dual roles of tumor promotion and tumor suppression in esophageal cancer (Zhang et al., 2024[342]; Cui et al., 2021[38]; Wu et al., 2022[291]; Chen et al., 2021[23]).

## Cervical Cancer

Cervical cancer is the fourth most common cancer in terms of female morbidity and mortality, and globally it is the most common type of cancer in 25 countries and the most common cause of cancer-related deaths in 37 countries (Bray et al., 2024[9]). The study of m6A modifications in cervical cancer has gradually increased in recent years and has been found to act mainly as tumor promoters (Mao et al., 2023[170]). However, METTL3 and METTL14 have also been found to be both anti-oncogenic factors in cervical cancer. A study found that METTL3 can inhibit the survival ability of cervical cancer cells and increase cisplatin sensitivity (Li et al., 2021[135]). METTL14 enhances sorafenib-induced ferroptosis through the PI3K/Akt signaling pathway also inhibits cervical cancer (Li et al., 2024[131]).

## Endometrial Cancer

Mortality from endometrial cancer has been on an upward trend since the mid-1990s and remains an important cause of cancer deaths in women (Siegel et al., 2022[214]). Up to now, there is still a relative paucity of explorations on m6A modifications in the field of endometrial cancer. It has been shown that m6A writers METTL3 inhibits the proliferation and migration of endometrial cancer cells and promotes the proliferation of CD8+ T cells (Zhan et al., 2023[334]). However, another study showed that METTL3 upregulates FGD5-AS1 expression through m6A modification, enhances chemoresistance in endometrial cancer cells, and promotes immune escape (Hao et al., 2024[73]). Similar to METTL3, WTAP exhibits both oncogenic and anti-oncogenic effects in endometrial cancer (Wang et al., 2024[243]; Li et al., 2021[133]). Differently, METTL14 decreases GPX4 mRNA stability through a YTHDF2-dependent mechanism, increases lipid peroxidation levels, and accelerates iron death in endometrial cancer, and thereby inhibits tumor progression (Wang et al., 2023[278]). m6A readers and erasers act primarily as tumor promoters in endometrial cancer. The IGF2BPs family was found to promote endometrial cancer progression by regulating cell proliferation and cancer cell stemness (Wang et al., 2024[243]; Zhang et al., 2021[344]; Shi et al., 2024[212]). For m6A erasers, it has been shown that FTO promotes endometrial cancer metastasis by activating the WNT signaling pathway (Zhang et al., 2021[345]). And ALKBH5 promotes endometrial cancer proliferation and invasion by eliminating the m6A modification of IGF1R (Pu et al., 2020[193]).

## Ovarian Cancer

Ovarian cancer is the seventh most common cancer among women in the world and the gynecologic cancer with the highest mortality rate, with a survival rate of 46 % at five years after diagnosis (Lheureux et al., 2019[121]). Recent studies have found that m6A regulators function primarily as cancer promoting factors in ovarian cancer. METTL3 inhibits CCNG2 expression by promoting the maturation of pri-microRNA-1246, which promotes ovarian carcinogenesis and metastasis (Bi et al., 2021[7]). In addition to METTL3, m6A writers VIRMA and RBM15 function as oncogenes in ovarian cancer (Yuan et al., 2023[326]; Gan et al., 2023[57]). For m6A readers, the IGF2BPs family was found to promote proliferation, metastasis, and immune escape in ovarian cancer, which in turn promotes tumor progression (Wang et al., 2023[264], 2024[254]; Li et al., 2024[126]). YTHDF1/2 also promotes ovarian cancer progression by regulating mRNA stability of downstream target molecules (Liu et al., 2020[157]; Hao et al., 2021[72]; Xu et al., 2021[303]; Sun et al., 2023[222]). Furthermore, m6A erasers ALKBH5 promote ovarian cancer invasion, lymph node metastasis, and cisplatin resistance by regulating EMT, FAK, and JAK2/STAT3 signaling pathways (Sun et al., 2023[222]; Xu et al., 2024[307]; Nie et al., 2021[178]). Contrary to the above effects, METTL16, YTHDC1, and FTO all exert anti-oncogenic effects in ovarian cancer (Li et al., 2023[124]; Wang et al., 2023[272]; Huang et al., 2020[90]).

## Pancreatic Cancer

With 511,000 new cases and 467,000 deaths in 2022, pancreatic cancer ranks sixth in cancer-related mortality and has one of the worst prognoses among malignant tumors (Bray et al., 2024[9]). In pancreatic cancer research, m6A modifications have been found to play a key tumor-promoting role. m6A modifications have been found to promote proliferation (Jin et al., 2024[114]; Chen et al., 2023[19]; Hu et al., 2022[84]), metastasis (Zhou et al., 2023[369]; Deng et al., 2021[44]), stem cell-like properties (Jin et al., 2024[114]; Chen et al., 2023[19]; Ouyang et al., 2024[183]), and chemotherapy resistance (Ouyang et al., 2024[183]; Lin et al., 2023[143]; Su et al., 2023[219]) in pancreatic cancer. m6A writers METTL3 mediates cigarette smoke-induced m6A modification of miR-25-3p, leading to activation of oncogenic AKT-p70S6K signaling in pancreatic cancer (Zhang et al., 2019[341]). METTL5 promotes c-Myc translation leading to pancreatic cancer progression (Huang et al., 2022[89]). METTL14 leads to decreased PERP levels through m6A modification, which in turn promotes pancreatic cancer proliferation and metastasis (Wang et al., 2020[258]). WTAPP1 binds WTAP mRNA and recruits the EIF3 translation initiation complex to promote WTAP translation, which enhances the activation of Wnt signaling and ultimately triggers the malignant phenotype of pancreatic cancer (Deng et al., 2021[44]). In addition, ZC3H13 and RBM15 also promote pancreatic cancer progression by regulating DNA damage repair and tumor immune infiltration in pancreatic cancer (Huang et al., 2022[87]; Wang et al., 2024[270]). Similarly, the m6A reader IGF2BPs family and YTHDF proteins also play a role in promoting pancreatic cancer through different pathways (Jin et al., 2024[114]; Hu et al., 2022[84]; Lin et al., 2023[143]; Wan et al., 2019[241]; Peng et al., 2023[190]; Chen et al., 2024[22]). And for m6A erasers, FTO mediates m6A modification of PDGFC and stabilizes its expression, leading to reactivation of the Akt signaling pathway and promoting pancreatic cancer cell growth (Tan et al., 2022[233]). In contrast, ALKBH5 prevents pancreatic cancer progression in an m6A-dependent manner by a different mechanism (Guo et al., 2020[66]; Zhang et al., 2022[351]; He et al., 2021[75]).

## Head and Neck Cancer

Approximately 90 % of head and neck cancer cases are head and neck squamous cell carcinoma (Liu et al., 2024[160]), with data for 2022 reporting 946,456 new cases and 482,001 deaths (Bray et al., 2024[9]), suggesting that it remains an important cause of cancer deaths. METTL3 enhances the stability and upregulates the expression of CDC25B mRNA, which activates the G2/M phase of the cell cycle and leads to malignant progression of head and neck squamous cell carcinoma (Guo et al., 2022[67]). METTL3 also promotes BMI1 translation in an IGF2BP1-dependent manner, which in turn promotes proliferation and metastasis in oral squamous cell carcinoma (Liu et al., 2020[153]). RBM15-mediated IGF2BP3-dependent m6A modification enhances TMBIM6 stability and leads to laryngeal squamous cell carcinoma progression (Wang et al., 2021[275]). METTL14 is recruited by RASAL2-AS1 and promotes the expression of LIS1, which in turn promotes the progression of head and neck squamous cell carcinoma (Rong et al., 2024[201]). IGF2BPs has also been found to play a promotional role in head and neck cancer. IGF2BP1 and IGF2BP3 are involved in recognition and stabilization of m6A-tagged HOXC10 mRNA leading to head and neck squamous cell carcinoma growth and metastasis (Zhou et al., 2024[367]). IGF2BP3 also regulates autophagy and promotes laryngeal squamous cell carcinoma progression by activating the TMA7-UBA2-PI3K pathway (Yang et al., 2023[315]). IGF2BP2 is activated by KLF7-regulated super-enhancer-driven transcription and promotes malignant progression in head and neck squamous cell carcinoma (Cai et al., 2024[11]). However, Liang et al. demonstrated that METTL14 inhibited oral squamous cell carcinoma progression by post-transcriptionally enhancing RB1CC1 expression in an IGF2BP2-dependent manner (Liang et al., 2023[141]).

## Leukemia

Leukemias are a group of cancers of the hematopoietic system that are the 11th most prevalent cancer and the 10th leading cause of cancer deaths worldwide (Miranda-Filho et al., 2018[173]). Published studies have demonstrated that m6A modifiers all exhibit pro-oncogenic effects in leukemia. Professor Kouzarides' team at the University of Cambridge discovered in 2017 that METTL3 is recruited by CEBPZ into the promoters of specific genes, leading to an increase in translation of genes such as SP1 to promote cell growth in acute myeloid leukemia (Barbieri et al., 2017[4]). In 2021, the team further demonstrated the effectiveness of using the highly efficient METTL3 inhibitor STM2457 to treat acute myeloid leukemia (Yankova et al., 2021[320]). METTL16 has also been found to exert its oncogenic effects by reprogramming branched-chain amino acid metabolism in acute myeloid leukemia (Han et al., 2023[71]). Wang et al. and Shen et al. also demonstrated that ALKBH5 is required for the development of acute myeloid leukemia and maintenance of leukemic stem cell function (Shen et al., 2020[206]; Wang et al., 2020[248]). Similarly, the m6A eraser FTO was found to act as an oncogenic agent in acute myeloid leukemia in 2017 (Li et al., 2017[139]), and the small molecule inhibitors FB23 and FB23-2 were found to inhibit the proliferation of acute myeloid leukemia cells *in vitro* and *in vivo* in 2019 (Huang et al., 2019[96]).

## Kidney Cancer

Kidney cancer is the 9th most common cancer in men and the 14th most common cancer in women, with clear cell carcinoma of the kidney being the most common (Stewart et al., 2022[217]), and the incidence of kidney cancer continues to increase at an annual rate of 1.5 % (Siegel et al., 2024[213]). The study of m6A modification in kidney cancer has increased significantly in recent years. Aberrant activation of FTO sensitizes renal clear cell carcinoma to BRD9 inhibitors (Zhang et al., 2021[336]), and FTO inhibits clear cell renal cell carcinoma through the PGC-1α signaling axis (Zhuang et al., 2019[378]). However, recent studies have also found that FTO-mediated autophagy promotes the progression of clear cell renal cell carcinoma by regulating SIK2 mRNA stability (Xu et al., 2022[310]). This implies that the role of FTO in clear cell renal cell carcinoma needs to be further investigated in depth. IGF2BP1/3 also found to promote kidney cancer progression. IGF2BP1 interacts with LINC01426 to regulate the CTBP1/miR-423-5p/FOXM1 axis and thus promotes clear cell renal cell carcinoma progression (Jiang et al., 2021[110]). IGF2BP3 stable LncRNA CDKN2B-AS1 drives malignancy in renal clear cell carcinoma through activation of NUF2 transcription (Xie et al., 2021[302]). In contrast, IGF2BP2 acts as a tumor suppressor in kidney cancer (Pan et al., 2022[185]; Ren et al., 2024[197]). YTHDF proteins also function as a tumor suppressor in kidney cancer (Liu et al., 2022[161]; Li et al., 2022[122]; Dai et al., 2024[40]).

## Melanoma

The incidence of melanoma is increasing by 2-3 % per year from 2015-2019 (Siegel et al., 2024[213]). 100,640 new diagnoses of cutaneous melanoma and 8,290 deaths are expected globally in 2014 (Siegel et al., 2024[213]). Recent studies have found that METTL3 localizes to mRNAs for m6A modification with the help of DHPS to drive melanoma (Guo et al., 2024[65]). ALKBH5 promotes cutaneous melanoma by mediating the downregulation of ABCA1 expression in an m6A-dependent manner (Wang et al., 2024[246]). METTL14 mediates m6A modification of RUNX2 to activate the Wnt/β-catenin signaling pathway and promote choroidal melanoma migration and invasion (Zhang et al., 2022[350]). However, a recent study published the opposite view, that METTL14 exerts tumor suppression in ocular melanoma by promoting the expression of the tumor suppressor FAT4 in a YTHDF1-dependent manner (Zhuang et al., 2023[377]). Furthermore, in 2024 Han et al. designed RM3, a peptide inhibitor specifically targeting the METTL 3/14 complex, which showed inhibitory effects on a variety of melanoma cell lines and exhibited a lower IC_50_ compared to STM2457 (Yankova et al., 2021[320]; Han et al., 2024[68]).

## Glioblastoma

Glioblastoma is the most common brain tumor, accounting for 45-50 % of all primary malignant brain tumors, and has a very poor prognosis (Grabiec et al., 2024[64]). Currently m6A modifications in glioblastoma are understudied and controversial. METTL3 has been found to promote glioblastoma proliferation and self-renewal induced by PDGF signaling (Lv et al., 2022[166]). Moreover, METTL3 and YTHDF1 can directly target ADAR1 transcripts, leading to elevated expression and tumor-promoting effects in glioblastoma (Tassinari et al., 2021[235]). On the contrary, the view of another study suggests that overexpression of METTL3 inhibits the growth and self-renewal of glioblastoma (Cui et al., 2017[37]).

In 2017, m6A demethylase ALKBH5 was found to promote tumorigenicity of glioblastoma stem-like cells by maintaining FOXM1 expression (Zhang et al., 2017[347]). Subsequent studies have also found that ALKBH5 and USP36 interact to maintain stem cell properties in glioblastoma and promote tumor progression (Chang et al., 2023[14]). The IGF2BPs family plays a pro-carcinogenic role by regulating glioma occurrence, progression and temozolomide resistance (Wang et al., 2015[265]; Cun et al., 2023[39]; Zhang et al., 2023[354]; Li et al., 2022[123]). Yet another study indicated that IGF2BP1 stabilizes circSPECC1 expression and promotes its encoding of the SPECC1-415aa protein to inhibit proliferation and metastasis of glioblastoma cells (Wei et al., 2024[281]). In addition, YTHDF protein is also a pro-carcinogenic factor for glioblastoma (Yarmishyn et al., 2020[321]; Dixit et al., 2021[48]; Lee et al., 2023[117]).

## Osteosarcoma

Osteosarcoma is the most common primary malignant bone tumor, with the highest incidence in children, adolescents, and the elderly population >60 years of age (Beird et al., 2022[5]). Five-year survival rate for patients with metastatic osteosarcoma is <20 % (Gill and Gorlick, 2021[61]). m6A writers positively regulate malignant progression in osteosarcoma. METTL3-mediated m6A modification of LINC00520 promotes glycolysis and resistance to cisplatin in osteosarcoma by inhibiting ubiquitination of ENO1 (Wei et al., 2024[284]). METTL14-mediated methylation enhances the translational efficiency of MN1 and promotes osteosarcoma progression and chemoresistance to all-trans retinoic acid (Li et al., 2022[127]). METTL16, WTAP, VIRMA and RBM15 also positively regulate osteosarcoma proliferation, invasion and migration by regulating PI3K/AKT, JAK2/STAT3 and aerobic glycolysis pathways (Cheng et al., 2024[32]; Chen et al., 2020[24]; Luo et al., 2023[163]; Yang et al., 2023[313]).

## Other Cancers

Gallbladder cancer is a common malignant tumor of the gastrointestinal tract characterized by high aggressiveness (Piovani et al., 2024[192]). In gallbladder cancer, m6A modification of TRPM2-AS by METTL3/14 is recognized by IGF2BP2 and promotes tumor angiogenesis through activation of the NOTCH1 signaling pathway (He et al., 2024[76]). Retinoblastoma is a childhood retinal cancer with about 8,000 cases worldwide (Cobrinik, 2024[36]). YTHDF1 promotes retinoblastoma growth by binding to and enhancing the stability of mRNAs from multiple oncogenes (Luo et al., 2023[165]). In lymphoma, YTHDF2 promotes tumorigenesis in diffuse large B-cell lymphoma by regulating ACER2-mediated ceramide metabolism in an m6A-dependent manner (Chen et al., 2024[25]). Tumorigenicity due to the interaction of the m6A reader YTHDC1 and the RNA helicase DDX5 has been identified in rhabdomyosarcoma (Dattilo et al., 2023[42]). VIRMA promotes proliferation, migration, invasion and chemoresistance to cisplatin in germ cell tumors (Miranda-Gonçalves et al., 2021[174]). METTL3 induces c-MYC expression in thymic epithelial tumor to promote tumor proliferation (Iaiza et al., 2021[97]).

## Recent Advances and Future Directions

Since the first discovery of m6A modification in the 1970s, a large number of studies targeting m6A have emerged, especially in the field of cancer. Currently, m6A modifications have been shown to regulate cancer occurrence and development by modulating different target molecules. Although m6A modifications have been shown to be involved in the biological processes of cancer, their role in cancer is not yet fully sufficient. Of particular note, current reports in the literature show that m6A modification regulators have both tumorigenic and anti-tumorigenic effects in the same tumor, or that their effects in cancer are controversial. The reason for this may be that m6A modification regulators mediate different downstream mechanisms by regulating the transcripts of different genes, which ultimately exert both oncogenic and anti-oncogenic effects in the same tumor. However, this controversy has somewhat interfered and hindered subsequent studies of m6A modifications in tumors. In response to this situation, future researchers need to study the epigenetic modification network of the m6A regulatory process in greater depth to provide clearer targets for targeted tumor therapy.

In recent years, as m6A modification research in the field of human cancer is increasing, more and more evidence indicates the feasibility of targeting m6A modification regulators and its potential to become an alternative therapy for cancer chemotherapy resistance (Zhou et al., 2023[365]). In 2019, Professor Yang's team at the University of Chinese Academy of Sciences discovered the significant inhibitory effect of the FTO inhibitor FB23-2 on the proliferation of human acute myeloid leukemia cells (Huang et al., 2019[96]). Professor Kouzarides' team at the University of Cambridge has demonstrated the efficacy of STM2457, a small molecule inhibitor of METTL3, in treating acute myeloid leukemia in 2021 (Yankova et al., 2021[320]). In 2024, Professor Shi's team at Hunan University in China designed a peptide inhibitor that specifically targets the METTL 3/14 complex, showing inhibitory effects on multiple melanoma cell lines (Han et al., 2024[68]). Although these advances are encouraging, no drugs targeting m6A modification regulators have yet entered clinical trials, but there is no denying that these discoveries lay the groundwork for targeting m6A modifications for the treatment of human cancers in the future. We believe that more extensive and in-depth exploration of the mechanism of m6A regulation of human cancers will be carried out in the future and provide better m6A therapeutic targets and facilitate the generation of more effective targeted therapeutic drugs.

## Conclusions

This paper reviews the biological regulation of m6A modification regulators in human cancers. m6A modification regulators can regulate oncogene/anti-oncogene expression, cancer occurrence, cancer cell proliferation, invasion, migration, angiogenesis, cancer cell stemness, and chemoresistance to regulate cancer progression. The existing problem is that the research on m6A modification in cancer is not sufficient, and its deeper regulatory mechanism in cancer and the crosstalk of various m6A modification regulators in cancer are not yet fully understood. 

In addition, although effective small molecule compounds targeting m6A modification regulators have emerged, more studies are needed to demonstrate the clinical efficacy of targeting m6A modifications.

## Notes

Liang Shang, Feng Cao (Department of General Surgery, Xuanwu Hospital, Capital Medical University, Beijing, China; E-mail: f.cao@xwhosp.org) and Fei Li (Department of General Surgery, Xuanwu Hospital, Capital Medical University, Beijing, China; E-mail: feili36@ccmu.edu.cn) contributed equally as corresponding author.

## Declaration

### Ethics approval and consent to participate 

Not applicable.

### Consent for publication 

All authors gave their consent for publication. 

### Availability of data and materials 

Not applicable.

### Competing interests 

No authors have any conflict of interest or competing interests to declare.

### Funding 

This work was supported by the National Natural Science Foundation of China (82470678), Beijing Natural Science Foundation (7242069), Beijing Hospital Administration Training Project (PX2023030), and Capital Medical Development and Research Special Project (Z201100005520090).

### Author contributions 

X.X and Z.F retrieved articles and wrote the manuscript. H.Z, Z.W, J.L and Y.J drawn figure and table. L.S, F.C and F.L supervised this manuscript. All authors read and approved the final manuscript.

### Acknowledgments 

None.

## Figures and Tables

**Table 1:**
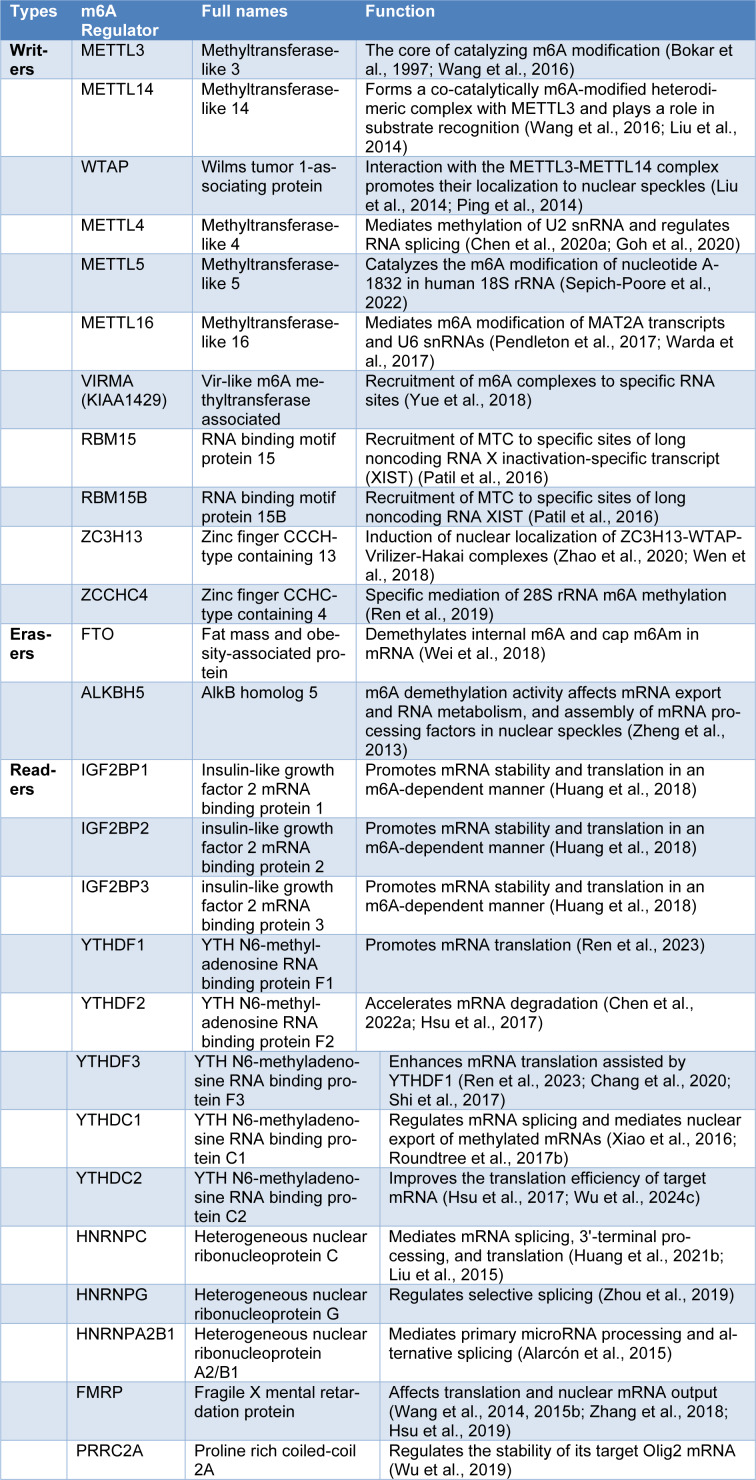
The function of m6A modification regulators

**Figure 1:**
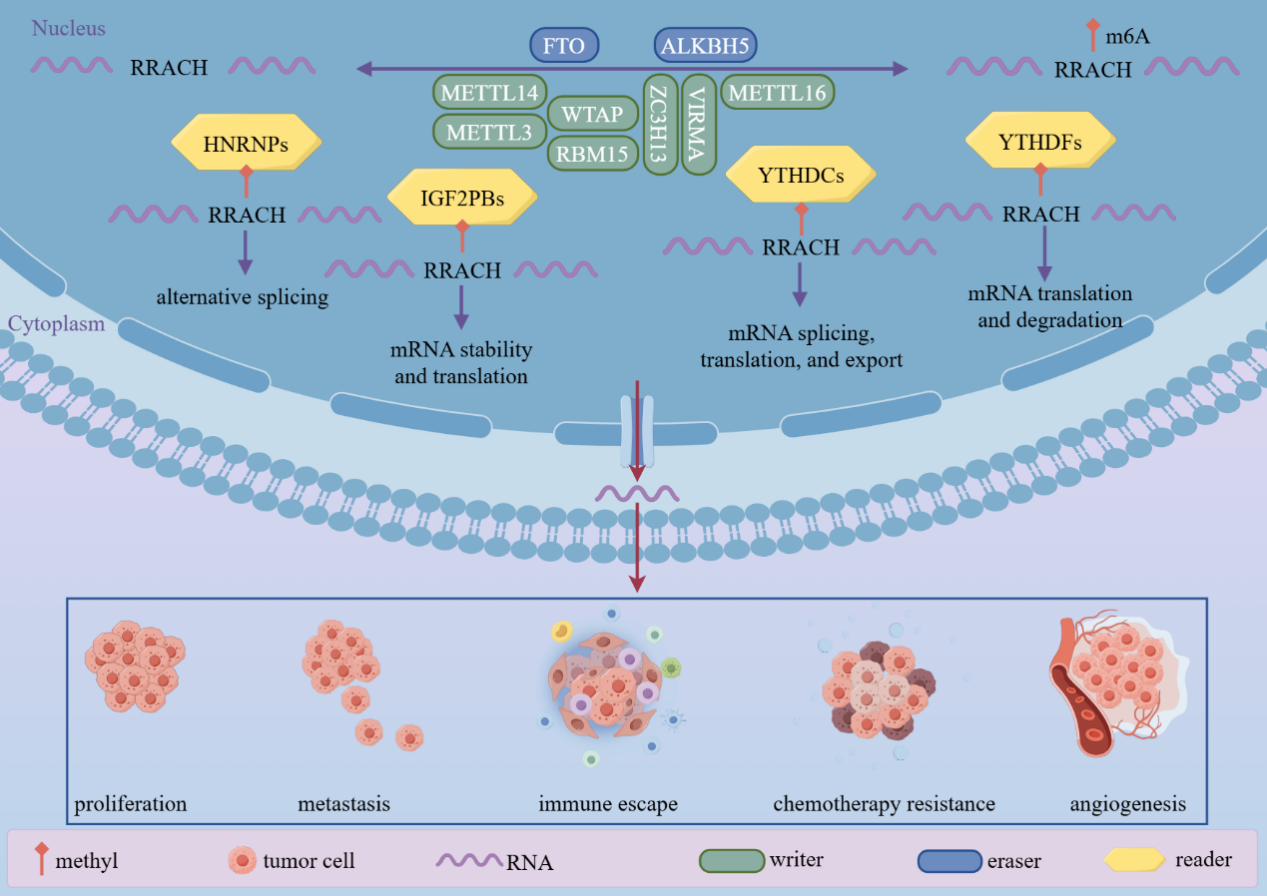
Graphical abstract: m6A modification regulators affect biological behaviors such as cancer proliferation, metastasis, immune escape, chemoresistance and angiogenesis by regulating m6A modifications of downstream target mRNAs.
